# Investigation of *Salmonella* Enteritidis outbreaks in South Africa using multi-locus variable-number tandem-repeats analysis, 2013-2015

**DOI:** 10.1186/s12879-017-2751-8

**Published:** 2017-10-02

**Authors:** Munyadziwa Muvhali, Anthony Marius Smith, Andronica Moipone Rakgantso, Karen Helena Keddy

**Affiliations:** 1 Centre for Enteric Diseases, National Institute for Communicable Diseases, Johannesburg, South Africa; 2 Faculty of Health Sciences, University of the Witwatersrand, Johannesburg, South Africa; 3 Outbreak Response Unit, Division of Public Health Surveillance and Response, National Institute for Communicable Diseases, Johannesburg, South Africa

**Keywords:** Non-typhoidal *Salmonella* (NTS), *Salmonella* Enteritidis, Multi-locus variable-number tandem-repeats analysis (MLVA), Variable-number tandem-repeat (VNTR), *Salmonella* outbreaks

## Abstract

**Background:**

*Salmonella enterica* serovar Enteritidis (*Salmonella* Enteritidis) has become a significant pathogen in South Africa, and the need for improved molecular surveillance of this pathogen has become important. Over the years, multi-locus variable-number tandem-repeats analysis (MLVA) has become a valuable molecular subtyping technique for *Salmonella,* particularly for highly homogenic serotypes such as *Salmonella* Enteritidis. This study describes the use of MLVA in the molecular epidemiological investigation of outbreak isolates in South Africa.

**Methods:**

Between the years 2013 and 2015, the Centre for Enteric Diseases (CED) received 39 *Salmonella* Enteritidis isolates from seven foodborne illness outbreaks, which occurred in six provinces. MLVA was performed on all isolates.

**Results:**

Three MLVA profiles (MLVA profiles 21, 22 and 28) were identified among the 39 isolates. MLVA profile 28 accounted for 77% (30/39) of the isolates. Isolates from a single outbreak were grouped into a single MLVA profile. A minimum spanning tree (MST) created from the MLVA data showed a close relationship between MLVA profiles 21, 22 and 28, with a single VNTR locus difference between them.

**Conclusions:**

MLVA has proven to be a reliable method for the molecular epidemiological investigation of *Salmonella* Enteritidis outbreaks in South Africa. These foodborne outbreaks emphasize the importance of the One Health approach as an essential component for combating the spread of zoonotic pathogens such as *Salmonella* Enteritidis.

## Background

*Salmonella* is a major cause of morbidity and mortality in children under the age of five in most developing countries worldwide [1–3]. The global human health impact of nontyphoidal *Salmonella* (NTS) is high, with an estimated 93.8 million illnesses, of which 80.3 million are reported to be foodborne related, and 155,000 deaths each year [4]. Human illness caused by *Salmonella enterica* serovar Enteritidis (*Salmonella* Enteritidis) has drastically increased worldwide, and by the 1980’s *Salmonella* Enteritidis had replaced *Salmonella enterica* serovar Typhimurium (*Salmonella* Typhimurium) as the primary cause of salmonellosis globally [5].

In developed countries, NTS usually causes self-limiting gastroenteritis with fewer numbers of deaths in humans compared to developing countries [6]. In Africa, NTS is commonly associated with invasive disease which leads to a high burden of morbidity and mortality [7–9]. In Africa, the burden of non-invasive *Salmonella* Enteritidis has not been established. However, it is estimated that *Salmonella* Enteritidis accounts for 33.1% of the total invasive NTS infections [10]. Despite global efforts to curb its spread *Salmonella* Enteritidis infections persist, causing an on-going challenge to the global health system.

In South Africa, laboratory-based surveillance of enteric bacteria for public health importance was initiated in 2003, by the Centre for Enteric Diseases (CED) at the National Institute for Communicable Diseases (NICD). The surveillance was mainly in response to the human immunodeficiency virus (HIV) epidemic in the country. During that period, the predominant invasive NTS serotypes were *Salmonella* Typhimurium and *Salmonella enterica* serovar Isangi [6, 11]. The introduction of highly active antiretroviral therapy (HAART) in 2004 showed a gradual decline in invasive salmonellosis, more especially in those serotypes that were associated with HIV infection such as *Salmonella* Typhimurium, whose association with HIV in Africa has been extensively described [12]. Even so, *Salmonella* Typhimurium remained the most common cause of salmonellosis in South Africa. However, in 2011 *Salmonella* Enteritidis became more prevalent and overtook *Salmonella* Typhimurium as the most commonly identified *Salmonella* serotype, and the overall number of *Salmonella* Enteritidis cases reported to the CED have increased [13, 14]. This increase is still inexplicable and it is independent of the HIV epidemic in the country.

Pulsed-field gel electrophoresis (PFGE) is still commonly used for the molecular subtyping of *Salmonella*. However, it lacks good discriminatory power in genetically homogeneous serotypes such as *Salmonella* Enteritidis. In most instances, it is unable to successfully discriminate outbreak from non-outbreak strains and in such cases, successful discrimination is only attained through the combination of intensive epidemiological, genotypic and phenotypic methods [15–17].

Over the years, multi-locus variable-number tandem-repeats analysis (MLVA) has become a useful molecular subtyping technique for *Salmonella* and it has shown good discriminatory power between *Salmonella* Enteritidis strains [17, 18]. This technique characterises strains based on size differences in amplified DNA fragments at various variable-number tandem-repeat (VNTR) loci regions, found in the genome of most bacterial species [19, 20].

Between the years 2013 and 2015, the CED received isolates from seven foodborne illness outbreaks, for further laboratory analysis. The outbreaks occurred within six provinces in South Africa namely; Gauteng (GA), Limpopo (LP), Mpumalanga (MP), Eastern Cape (EC), Free State (FS) and KwaZulu-Natal (KZN). In this paper, we describe the use of MLVA in the molecular investigation of isolates from these outbreaks.

## Methods

### Outbreaks and notification

A foodborne illness outbreak is defined as any food poisoning incident involving two or more individuals that are epidemiologically linked to a common food/beverage source. Foodborne outbreaks are reported to the Outbreak Response Unit (ORU) of the NICD, which provides technical support for outbreak investigation and control in South Africa. The ORU works in close association with the CED linking molecular data with epidemiological data from the outbreak investigation [21]. Between 2013 and 2015, seven suspected outbreaks were reported to ORU, in which NTS was the implicated pathogen.

### Receiving and processing of outbreak isolates at the CED

The CED serves as a reference centre for human enteric pathogens in South Africa, including those identified in outbreaks. During outbreak investigations, the CED receives suspected outbreak-associated isolates from food and environmental (public health) laboratories and the clinical diagnostics microbiology laboratory involved. *Salmonella* isolates from seven suspected outbreaks were sent to CED for further characterisation. Isolate identification was confirmed using the Vitek®2 60 system (bioMérieux, Durham, United States of America) and serotyping (White-Kauffman-Le Minor scheme) [22].

### Crude genomic DNA extraction from bacteria

Crude DNA was extracted from a pure overnight culture of *Salmonella* Enteritidis by inoculating a small loopful of bacterial culture in autoclaved TE buffer (10 mM Tris, 1 mM EDTA; pH 8.0) and boiling the suspension at 95 °C for 25 min (min). The suspension was centrifuged at 13200 rpm for 3 min to pellet the cellular debris and 20 μl of the supernatant was diluted in 80 μl of autoclaved TE buffer (pH 8.0).

### MLVA

A previously described MLVA technique containing five VNTR loci (SENTR7-SENTR5-SENTR6-SENTR4-SE-3) for *Salmonella* Enteritidis was used in this study (Table 1) [15]. A multiplex PCR was performed to amplify the five VNTR loci regions. Each PCR run and subsequent MLVA analysis always included a negative control (reaction tube with no DNA added) and a positive control. The positive control included the analysis of a well-validated *Salmonella* Enteritidis isolate; this isolate was well validated previously and consistently showed a VNTR loci allele size pattern (123–292–184-112-306).

**Table 1:** MLVA loci and PCR primer sequences

Target gene locus	PCR primer	Primer sequence (5′ to 3′)	Expected fragment sizes (bp)	VNTR repeat length (bp)	VNTR references
SENTR7	SENTR7-F	6FAM-ACGATCACCACGGTCACTTC	117–135	9	[15]
	SENTR7-R	CGGATAACAACAGGACGCTTC			
SENTR5	SENTR5-F	6FAM-CACCGCACAATCAGTGGAAC	235–301	6	[15]
	SENTR5-R	GCGTTGAATATCGGCAGCATG			
SENTR6	SENTR6-F	NED-ATGGACGGAGGCGATAGAC	173–236	7	[15]
	SENTR6-R	AGCTTCACAATTTGCGTATTCG			
SENTR4	SENTR4-F	VIC-GACCAACACTCTATGAACCAATG	112–147	7	[15]
	SENTR4-R	ACCAGGCAACTATTCGCTATC			
SE-3	SE-3-F	VIC-CAACAAAACAACAGCAGCAT	308–320	12	[15]
	SE-3-R	GGGAAACGGTAATCAGAAAGT			

A Qiagen multiplex PCR kit (Qiagen, Hilden, Germany) was used for the PCR. Each 25 μl reaction contained 12.5 μl of the Qiagen master mix, 2.5 μl Qiagen Q-solution, 1 μM of each fluorophore-labelled forward and reverse primer, and 50 ng of DNA template (1 μl of crude genomic DNA preparation). The PCR cycling conditions included: denaturation at 95 °C for 15 min, followed by 35 cycles of 60 s (sec) at 94 °C, 90 s at 55 °C, 90 s at 72 °C, and a final extension at 72 °C for 10 min. The PCR amplicons were diluted 2:198 in PCR grade water and 1 μl of the dilution was combined with 0.2 μl of GeneScan 600 LIZ Standard v2.0 (Applied Biosystems, Foster City, USA) and 12 μl of Hi-Di formamide (Life Technologies, Warrington, UK).

The fragment size of each VNTR locus was determined by capillary electrophoresis on the Applied Biosystems 3500 Genetic Analyzer (Applied Biosystems). Data was analysed using the GeneMapper Software version 4.1 (Applied Biosystems) and is briefly described as follows. The DNA fragments were automatically allocated to length bins and the VNTR loci alleles were assigned based on the bin fragment sizes. The VNTR loci allele sizes were captured into the BioNumerics Software version 6.5 (Applied Maths, Sint-Martens-Latem, Belgium) as character values. The VNTR loci allele sizes were used to assign MLVA profile numbers. A single VNTR locus difference resulted in a new MLVA profile being defined (e.g. 123, 268, 184, 112, 318_ MLVA profile 1 and 123, 262, 184, 112, 318_ MLVA profile 2). A dendrogram was constructed by the UPGMA method, using the categorical coefficient with a 0 tolerance and a minimum spanning tree (MST) was constructed using the MST categorical coefficient.

## Results

### Isolates and outbreaks

From 2013 to 2015, the CED received 39 isolates associated with seven reported foodborne illness outbreaks. A total of 38/39 (97%) isolates (isolated from stool samples) were obtained from human cases. One isolate was obtained from each human case. Of the 39 isolates, 1/39 (3%) isolate was obtained from a food sample. All 39 isolates from the seven outbreaks were confirmed to be *Salmonella* Enteritidis through serotyping.

### Outbreak 1

Outbreak 1 occurred in the KZN province during May 2013. Two people became ill after consuming meat (the liver) from a goat that had died from illness. Three isolates were received (two human isolates and one goat meat isolate). The outbreak isolates were analysed with MLVA retrospectively and all the isolates belonged to MLVA profile 22 (Tables 2 and 3).

**Table 2:** Detailed summary of the seven *Salmonella* Enteritidis outbreaks

Outbreak	Province^a^	Outbreak date	No. of cases	No. of patient deaths	No. of patients admitted	Clinical samples collected	Food samples tested	Food testing results	No. of isolates tested at CED	CED test results
1	KZN	May 2013	2	0	0	Stool (*n* = 2)	Goat meat (*n* = 1)	*Salmonella* Enteritidis	3	*Salmonella* Enteritidis
2	MP	November 2013	unknown	unknown	unknown	unknown	unknown	unknown	3	*Salmonella* Enteritidis
3	LP	January 2014	65	0	8	Stool (*n* = 8)	Chicken (*n* = unknown)	unknown	3	*Salmonella* Enteritidis
4	MP	July 2014	46	0	6	Stool (*n* = 12) Rectal swabs (*n* = 2)	unknown	unknown	14	*Salmonella* Enteritidis
5	FS	November 2014	80	unknown	6	unknown	unknown	unknown	3	*Salmonella* Enteritidis
6	EC	December 2014	unknown	unknown	unknown	unknown	unknown	unknown	10	*Salmonella* Enteritidis
7	GA	October 2015	4	0	4	Stool (*n* = 4)	unknown	unknown	3	*Salmonella* Enteritidis

Provinces^a^: Gauteng (GA), Limpopo (LP), Mpumalanga (MP), Eastern Cape (EC), Free State (FS) and KwaZulu-Natal (KZN)

*n* = total number

**Table 3:** Summary of the outbreaks MLVA data

Outbreak	Province^a^	VNTR loci fragment sizes (SENTR7-SENTR5-SENTR6-SENTR4-SE-3)	MLVA profile
Outbreak 1	KZN	123–262–184-112-318	22
Outbreak 2	MP	123–262–184-112-318	22
Outbreak 3	LP	123–268–184-112-318	28
Outbreak 4	MP	123–268–184-112-318	28
Outbreak 5	FS	123–268–184-112-318	28
Outbreak 6	EC	123–268–184-112-318	28
Outbreak 7	GA	123–274–184-112-318	21

Province^a^: Gauteng (GA), Limpopo (LP), Mpumalanga (MP), Eastern Cape (EC), Free State (FS) and KwaZulu-Natal (KZN)

### Outbreak 2

Outbreak 2 occurred in the MP province in November 2013. The outbreak was associated with food poisoning. However, no further details were provided about the outbreak. Three human isolates were received from the outbreak. The outbreak isolates were analysed with MLVA retrospectively and all the isolates belonged to MLVA profile 22 (Tables 2 and 3).

### Outbreak 3

Outbreak 3 occurred in the LP province in January 2014. This foodborne outbreak occurred in a lodge. Sixty-five people were affected, eight of whom were admitted to hospital in critical condition. Three human isolates were received from the outbreak. The outbreak isolates were analysed with MLVA retrospectively and all the isolates belonged to MLVA profile 28 (Tables 2 and 3).

### Outbreak 4

Outbreak 4 occurred in the MP province in July 2014. The outbreak was associated with food prepared for a funeral. Forty-six people were affected, six of whom were children who were admitted to hospital in critical condition. Fourteen human isolates were received from the outbreak. The outbreak isolates were analysed with MLVA retrospectively and all the isolates belonged to MLVA profile 28 (Tables 2 and 3).

### Outbreak 5

Outbreak 5 occurred in the FS province in November 2014. The outbreak was associated with food prepared for a function in a mine. Eighty people were affected, six of whom were hospitalized. Three human isolates were received from the outbreak. All isolates belonged to MLVA profile 28 (Tables 2 and 3).

### Outbreak 6

Outbreak 6 occurred in the EC province in December 2014. The outbreak occurred in a TB hospital. However, no further details were provided about the outbreak. Ten human isolates were received from the outbreak. All isolates belonged to MLVA profile 28 (Tables 2 and 3).

### Outbreak 7

Outbreak 7 occurred in the GA province in October 2015. The outbreak was in a private residence, where a mother had cooked chicken feet for dinner. Four children were affected (age 4, 7, 8 and 11). Three human isolates were received from the outbreak. All isolates belonged to MLVA profile 21 (Tables 2 and 3).

### MLVA data

**Fig. 1:**
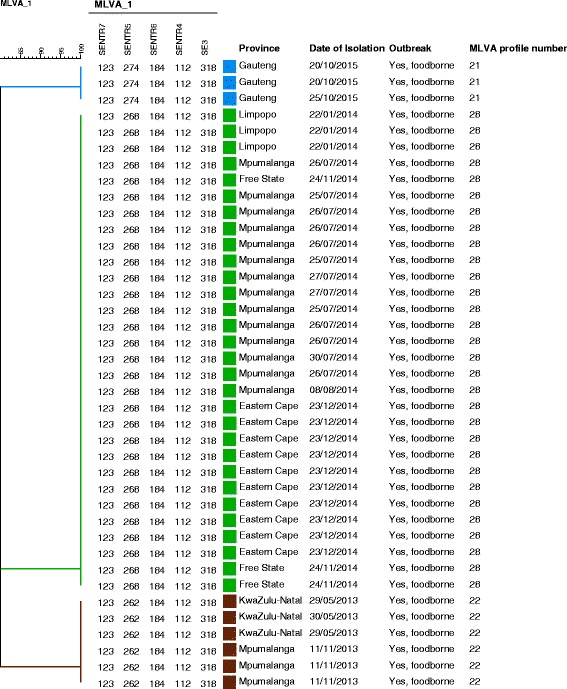
MLVA dendrogram of the *Salmonella* Enteritidis outbreak isolates. MLVA profile 21 is highlighted in blue, MLVA profile 28 in green and MLVA profile 22 in brown

The seven outbreaks showed a total of three different MLVA profiles. All isolates within an outbreak always showed an identical MLVA profile (Fig. 1). MLVA profile 21 (123_274_184_112_318) was present in 3/39 (7.7%) isolates; all the isolates were from outbreak 7 (GA). MLVA profile 22 (123_262_184_112_318) was present in 6/39 (15.3%) isolates; three isolates from outbreak 1 (KZN) and three isolates from outbreak 2 (MP). MLVA profile 28 (123_268_184_112_318) accounted for 77% (30/39) of the total outbreak isolates. This MLVA profile contained three isolates from outbreak 3 (LP), 14 isolates from outbreak 4 (MP), three isolates from outbreak 5 (FS) and 10 isolates from outbreak 6 (EC). Consequently, MLVA profile 28 was the most predominant MLVA profile, followed by MLVA profile 22 and 21 respectively.

## Discussion

For many years, PFGE was considered the gold standard for subtyping of salmonellae. It was deemed a useful method for outbreak investigations in support of phage typing, when further strain discrimination was required. However, *Salmonella* Enteritidis has limited heterogeneity (lacks genetic variation), thus making discrimination beyond phage typing challenging [15]. The application of MLVA to a wide variety of bacterial species including *Salmonella* species showed that it was more discriminatory compared to other available molecular subtyping methods [17].

**Fig. 2:**
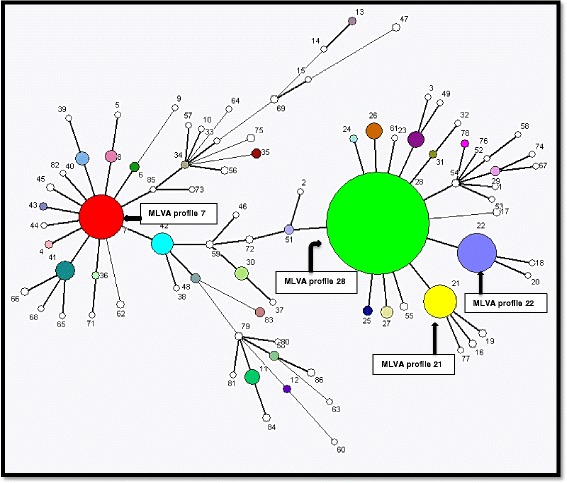
MLVA MST of *Salmonella* Enteritidis isolates in the CED database. The circle (node) size represents the MLVA profile and the size of the node represents the number of isolates in each MLVA profile (the smaller node the fewer the number of isolates in the MLVA profile). MLVA profiles are connected by branches and the thickness of the branch indicates how many VNTR loci differences are between the connected MLVA profiles. The thick solid lines connect MLVA profiles (nodes) that differ by one VNTR locus and thin solid lines connect MLVA profiles that have two VNTR loci difference. The distance between the MLVA profiles represents the genetic divergence between two neighbouring MLVA profiles

The CED currently does MLVA for *Salmonella* Enteritidis isolates from the GA and Western Cape (WC) Provinces. The analysis of the GA and WC *Salmonella* Enteritidis isolates using MLVA was initiated in 2013. The VNTR loci allele sizes obtained from these isolates were used to establish a *Salmonella* Enteritidis MLVA profile number database. In the established CED MLVA database, 84 MLVA profiles have been determined from 1221 human isolates, obtained from various body sites (manuscript in preparation-unpublished data). Of the 84 MLVA profiles, four notable MLVA profiles (MLVA profiles 28, 7, 22 and 21) have been identified (Fig. 2).

In this current study, MLVA profile 28 accounted for majority of the outbreaks (5/7 outbreaks), thus showing that it may be highly propagative within South Africa. Furthermore, MLVA profile 28 accounts for a large number of isolates in the established CED MLVA database. MLVA profiles 21 and 22 are also some of the common MLVA profiles in the CED MLVA database. Further analysis of the MST shows that MLVA profiles 21 and 22 are closely related to MLVA profile 28, with a single VNTR locus difference between them (Fig. 2). This difference was observed in VNTR locus SENTR5. High variation (high number of alleles) within the SENTR5 locus was previously described by Malorny et al. [23], whereby SENTR5 had the second highest number of alleles in their study (10 alleles), preceded by SENTR6 (11 alleles).

In our study, SENTR5 helped show the genetic variation present within these closely related MLVA profiles. Such close relation suggests that minor changes may have occurred between these MLVA profiles and that they may have shared characteristics, which enable them to spread effectively throughout the country and cause more outbreaks, compared to other MLVA profiles. However, more outbreaks need to be analysed with MLVA, supplemented by whole genome sequencing in order to investigate such possible shared characteristics. Nonetheless a similar event has been reported previously in a study by Slinko et al., [20] on an outbreak of *Salmonella* Typhimurium in Brisbane Australia. Slinko et al., [20] found that outbreaks caused by the STm197 strain had produced several closely related MLVA profiles, which had caused outbreaks in many restaurants throughout the city for over two months.

**Fig. 3:**
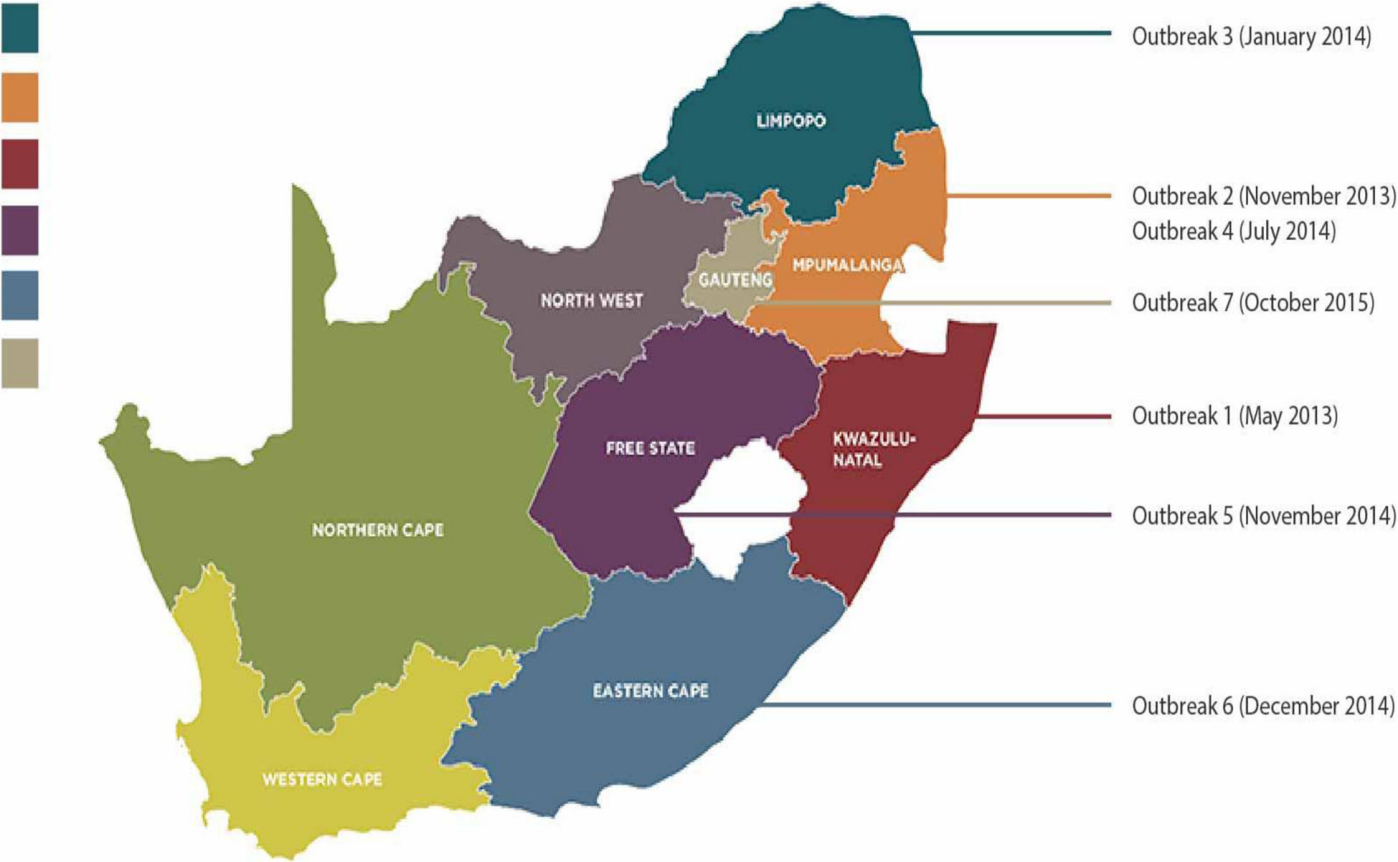
South African map, illustrating the geographical regions of the seven *Salmonella* Enteritidis outbreaks, from the years 2013–2015

Although the three MLVA profiles in our study were closely related, geographical and epidemiological analysis of the seven outbreaks does not indicate any possible links between the outbreaks. Majority of the outbreaks occurred several months apart, except for outbreak 5 (Free State Province) and outbreak 6 (Eastern Cape Province), which occurred days from each other. However, they could not be linked epidemiologically (Fig. 3).

Numerous studies have applied MLVA in the analysis of *Salmonella* Enteritidis in different parts of the world, and many studies have applied it in outbreak investigations [15]. However, many have remained doubtful about the stability of VNTR’s during an outbreak. It is assumed that VNTR’s may evolve rapidly, thereby producing multiple MLVA profiles during an outbreak [18]. Studies by Boxrud et al., [17] and Malorny et al., [23] analysed the stability of *Salmonella* Enteritidis VNTR’s during an outbreak and found that the VNTR’s had remained stable during outbreak investigations. Our current study was able to show that MLVA can be used as a molecular epidemiological tool for the investigation of outbreaks associated with *Salmonella* Enteritidis. MLVA was able to group all isolates from a single outbreak into a single MLVA profile; indicating the stability of the VNTR’s during an outbreak. Furthermore, MLVA has faster turnaround times compared to PFGE. It is this MLVA advantage that enabled the reporting of strain relatedness results from outbreaks 5–7 (outbreaks were analysed with MLVA in real time) to the relevant personnel more quickly, thus enhancing the public health interventions.

In this study, outbreak 1_ (KZN) included an isolate from goat meat. Such a finding emphasizes the role food animals have in the spread of zoonotic pathogens (such as *Salmonella* Enteritidis) to the human population [24]. Therefore, it is crucial that human and animal health organisations work together to reduce pathogen transmission. More so, this also emphasizes the importance of testing food items implicated in an outbreak, in order to determine the source and cause of infection and disease.

Our study had a number of limitations. Firstly, MLVA was performed on our outbreak isolates long before the recent publication of Peters et al., [25], which described the validation of a reference/calibration set of strains for MLVA of *Salmonella* Enteritidis. Currently, we don’t have access to this reference/calibration set of strains, as we were never invited to participate in the multi-laboratory validation study. Therefore, we were not able to normalize our MLVA data to obtain exact repeat numbers and describe MLVA profiles in the format of numbers of repeats. Therefore, currently inter-laboratory comparison of our MLVA data with other laboratories will be a challenge. Secondly, the epidemiological and clinical data of the outbreaks were not complete. This limited our ability to further analyse the link between the molecular data and epidemiological data. Lastly, all the outbreaks were foodborne related, but only two outbreaks (outbreak 1 and outbreak 3) had food items tested. However, results from outbreak 3 food items is unknown. This limited our ability to compare human isolates to the non-human (food) isolates.

## Conclusions

In conclusion, MLVA has shown to be a reliable method for the molecular epidemiological investigation of *Salmonella* Enteritidis outbreaks in South Africa. Our findings emphasize the need for analysis of *Salmonella* Enteritidis isolates from different provinces in South Africa, in order to investigate the circulating strains (and MLVA profiles) in each province and to investigate their potential to cause outbreaks. Furthermore, this study emphasizes the importance of using the One Health approach; combining food, animal and human testing in order to curb the spread of foodborne zoonotic diseases within the country. In association with the current epidemiological surveillance programs, studies such as this can provide valuable information for the development of public health strategies to minimize or control the risk of outbreaks associated with *Salmonella* Enteritidis in South Africa.

## Abbreviations

CED: Centre for Enteric Diseases
EC: Eastern Cape
FS: Free State
GA: Gauteng
HAART: Highly active antiretroviral therapy
HIV: Human immunodeficiency virus
KZN: KwaZulu-Natal
LP: Limpopo
MLVA: Multi-locus variable-number tandem-repeats analysis
MP: Mpumalanga
MST: Minimum spanning tree
NICD: National Institute for Communicable Diseases
NTS: Non-typhoidal *Salmonella*
ORU: Outbreak Response Unit
PFGE: Pulsed-field gel electrophoresis
*Salmonella* Enteritidis: *Salmonella enterica* serovar Enteritidis
VNTR: Various variable-number tandem-repeat
WC: Western Cape
